# Progression of coronary artery atherosclerosis in rheumatoid arthritis: comparison with participants from the Multi-Ethnic Study of Atherosclerosis

**DOI:** 10.1186/ar4314

**Published:** 2013-09-25

**Authors:** Cecilia P Chung, Jon T Giles, Richard A Kronmal, Wendy S Post, Allan C Gelber, Michelle Petri, Moyses Szklo, Robert Detrano, Matthew J Budoff, Roger S Blumenthal, Pamela Ouyang, David Bush, Joan M Bathon

**Affiliations:** 1 Division of Rheumatology, Johns Hopkins University School of Medicine, Baltimore, MD, USA; 2 Division of Rheumatology, Columbia University, New York, NY, USA; 3 Division of Cardiology, Johns Hopkins University School of Medicine, Baltimore, MD, USA; 4 Department of Biostatistics, University of Washington, Seattle, WA, USA; 5 Department of Epidemiology, Johns Hopkins University Bloomberg School of Public Health, Baltimore, MD, USA; 6 Department of Radiological Sciences, University of California at Irvine, Irvine, CA, USA; 7 Los Angeles Biomedical Research Institute at Harbor-UCLA, Torrance, CA, USA; 8 Vanderbilt University School of Medicine, Nashville, USA

## Abstract

**Introduction:**

In cross-sectional studies, patients with rheumatoid arthritis (RA) have higher coronary artery calcium (CAC) than controls. However, their rate of progression of CAC and the predictors of CAC progression have heretofore remained unknown.

**Methods:**

Incidence and progression of CAC were compared in 155 patients with RA and 835 control participants. The association of demographic characteristics, traditional cardiovascular risk factors, RA disease characteristics and selected inflammatory markers with incidence and progression of CAC were evaluated.

**Results:**

The incidence rate of newly detected CAC was 8.2/100 person-years in RA and 7.3/100 person-years in non-RA control subjects [IRR 1.1 (0.7-1.8)]. RA patients who developed newly detectable CAC were older (59±7 vs. 55±6 years old, p=0.03), had higher triglyceride levels (137±86 vs. 97±60 mg/dL, p=0.03), and higher systolic blood pressure (129±17 vs. 117±15 mm Hg, p=0.01) compared to those who did not develop incident CAC. Differences in blood pressure and triglyceride levels remained significant after adjustment for age (p<=0.05). RA patients with any CAC at baseline had a median rate of yearly progression of 21 (7–62) compared to 21 (5–70) Agatston units in controls. No statistical differences between RA progressors and RA non-progressors were observed for inflammatory markers or for RA disease characteristics.

**Conclusions:**

The incidence and progression of CAC did not differ between RA and non-RA participants. In patients with RA, incident CAC was associated with older age, higher triglyceride levels, and higher blood pressure, but not with inflammatory markers or RA disease characteristics.

## Introduction

Patients with rheumatoid arthritis (RA) die prematurely [1] and the leading cause of death is coronary artery disease [2-7].

High resolution computed tomography is a non-invasive technique that allows the identification and quantification of coronary artery calcium (CAC). Prior studies have shown that CAC scores correlate with the presence and extent of coronary plaque [8] and its presence is associated with a higher risk of coronary heart disease [9-12].

Our previous research showed that patients with RA have higher CAC scores than control subjects [13,14]. These studies indicated that there was an association between higher CAC scores with sedimentation rate, smoking and a composite score of disease activity and severity. However, these associations were established in cross-sectional analyses; thus, they did not allow assessment of temporal sequence, which would further support causality.

The rate and predictors of progression of coronary atherosclerosis in RA are unknown. Therefore, we set out to test the hypotheses that: (1) subclinical coronary artery atherosclerosis, as measured by CAC, progresses more rapidly in patients with RA than in controls from a population based study; and (2) in RA subjects measures of disease activity, disease damage, inflammation and traditional cardiovascular risk factors are independent predictors of higher incident rates and progression of coronary artery atherosclerosis.

## Methods

**Figure 1:**
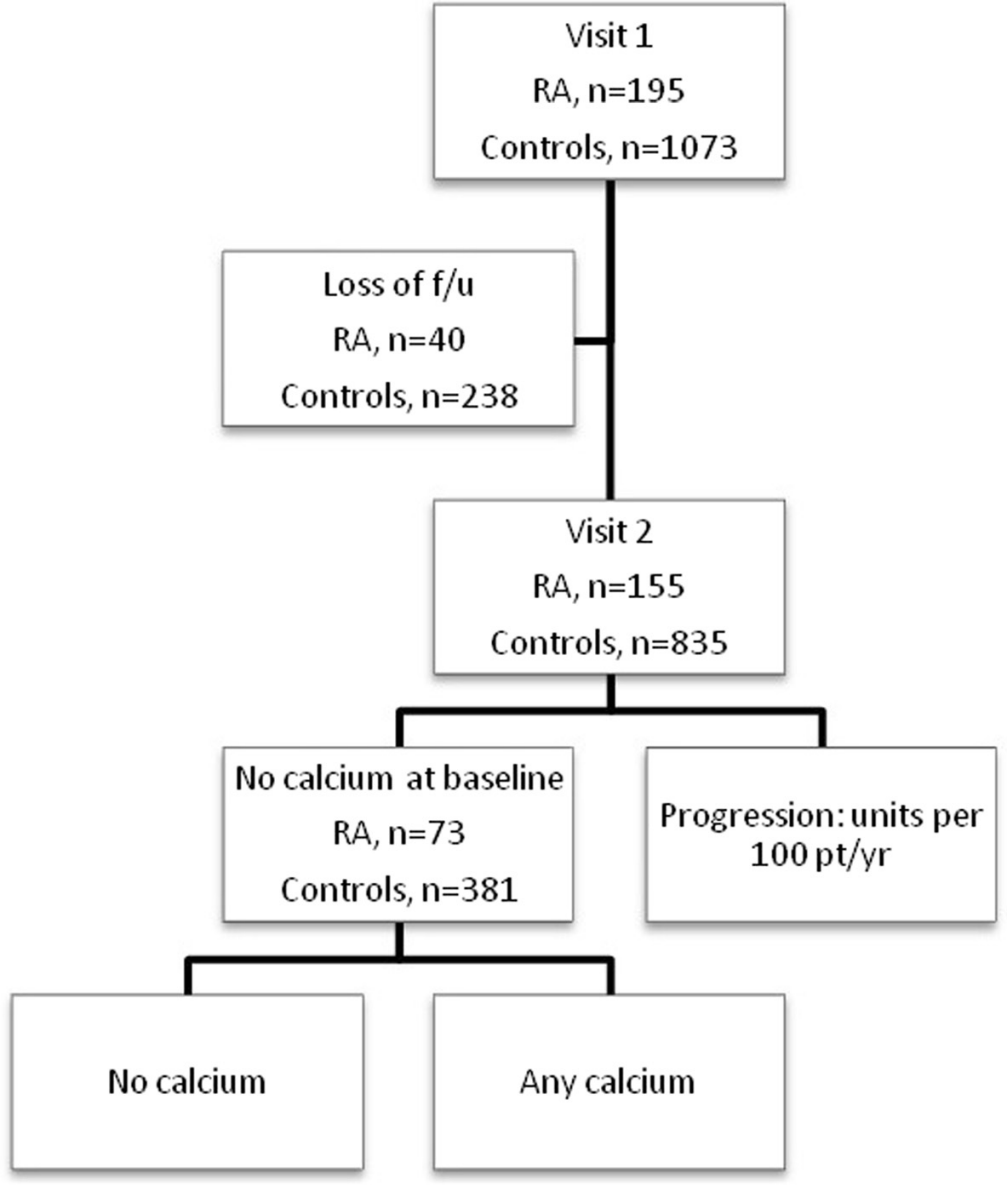
**Study design and disposition of participants.** f/u, follow-up; pt/yr, patient-years; RA, rheumatoid arthritis.

This is a prospective study of patients with RA and control subjects from two cohorts that followed similar protocols, the Evaluation of Subclinical Cardiovascular Disease and Predictors of Events in Rheumatoid Arthritis (ESCAPE RA) cohort [14-16] and the Multi-Ethnic Study of Atherosclerosis (MESA) [17]. The study design is summarized in Figure 1.

### Cohorts

The ESCAPE RA cohort was assembled to study the prevalence and progression of atherosclerosis and to identify risk factors for prevalent and progressive subclinical cardiovascular disease in patients with RA. Details of this cohort have been described before [14]. In brief, the cohort was assembled in the greater Baltimore area. Eligibility criteria included fulfillment of the 1987 American College of Rheumatology classification criteria for RA [18] and age 45 through 84 years. Major exclusion criteria were: (1) self-reported history of physician-diagnosed myocardial infarction, heart failure, coronary artery revascularization, peripheral arterial disease, implanted pacemaker or defibrillator devices and current atrial fibrillation; (2) weight exceeding 300 pounds; and (3) computerized tomographic (CT) scan of the chest within six months prior to study enrollment. A total of 197 patients were enrolled in this study from October 2004 through May 2006. Of these, 195 patients completed the initial evaluation, including assessment for the presence of CAC at baseline. Further, 155 patients had a three-year follow-up visit including a second CAC measurement. These 155 patients constituted the RA study sample for the present analyses.

Control subjects were part of the MESA (Multi-Ethnic Study of Atherosclerosis), a population-based cohort assembled in 2000 to 2002 to study the prevalence, risk factors and progression of subclinical atherosclerosis. Details of the study design have been published [17]. In brief, individuals were enrolled if they were 45 through 84 years of age and did not report a past history of a physician-diagnosed cardiovascular event. Eight hundred and thirty-five participants from the Baltimore Field Center who completed a follow-up evaluation, had CT evaluation at baseline and follow-up and were not taking any disease modifying anti-rheumatic drug, constituted the comparison group for these analyses. The study was approved by the Johns Hopkins Hospital Institutional Review Board and MESA, with all participants providing informed consent prior to enrollment.

### Study outcomes

CAC was ascertained with the use of cardiac CT using a multidetector row computed tomography (MDCT) system [19]. Scans were transmitted electronically to the MESA CT reading center where calcium scores were quantified using the methods described by Agatston [20]. Scoring of scans was blinded to the group allocation (RA and control).

Patients with RA had their second CAC measurement after an average of 3.2 (range: 2.2 to 4.2) years. In MESA participants, the second CAC scan was obtained on 391 participants of the MESA cohort during visit 2 and on 444 during visit 3. The mean follow-up time to repeat scan in controls was 2.3 (range: 0.9 to 4.6) years.

Given that CAC scores are highly skewed and that about half of the participants had a calcium score of zero at baseline, two pre-specified outcomes for subgroup analyses were defined following the design previously described by Kronmal *et al*. [21]:

– Incidence of CAC: all participants with a CAC score of zero at baseline were included in the analysis of incident CAC. The incident case definition required progression to a positive calcium score (≥1) over time.

– Progression of CAC: restricted to those participants with any detectable CAC at baseline.

### Study covariates

Demographic and clinical characteristics, cardiovascular risk factors, serum/plasma concentrations of inflammatory markers in RA patients and controls were collected following similar protocols.

### Clinical evaluation of cardiovascular risk factors

– Blood pressure (BP) was measured three times while individuals were sitting. The average of the last two measurements was used in the analysis. Hypertension was defined by systolic BP ≥140 mmHg, diastolic BP ≥90 mm Hg, or antihypertensive drug use.

– Diabetes was defined as a fasting glucose ≥126 mg/dL or use of anti-diabetic medications.

– Smoking status was ascertained by self-report.

### Laboratory methods

Fasting sera and plasma were separated by centrifugation and stored at -70°C. Total and high density lipoprotein (HDL) cholesterol, triglyceride, C-reactive protein (CRP), IL-6 and fibrinogen were measured at the MESA core laboratory, the Laboratory for Clinical Biochemistry Research (University of Vermont). In patients with RA, anti-cyclic citrullinated peptide (anti-CCP) antibodies and rheumatoid factor (RF) were measured as previously described [14]. Low density lipoprotein (LDL) cholesterol was calculated using the Friedewald equation [19].

### RA disease characteristics

The number of tender and swollen joints was ascertained and disease activity (DAS28) calculated using the 28-joint count and CRP [22]. Radiographic damage was quantified by the Sharp score and functional capacity was determined by the Health Assessment Questionnaire (HAQ) [23].

Anti-CCP antibody and RF greater than or equal to 60 and 40 units, respectively, met the definition of seropositivity.

The presence of HLA associated RA susceptibility alleles (the so-called 'shared epitope’) was defined as the presence of alleles QKRAA, QRRAA, QRAAA and RRRAA at positions 70 to 74 of Exon 2 of HLA-DRB1 using Allele SEQR HLA-DRB1 SBT kits (Abbott Molecular, Inc., Des Plaines, IL, USA). Radiographs of the hands and feet were scored using the Sharp-van der Heijde method [24] by a single, trained radiologist. As previously described, there were five subjects with incomplete radiographic assessments, in whom the scores were imputed from the available data [25].

### Statistical methods

For all variables, means and standard deviations summarize normally distributed data. For variables with skewed distribution, the data are presented as medians and interquartile ranges (IQRs). Clinical and laboratory data ascertained longitudinally are expressed as baseline and as average over follow-up. Categorical variables are presented as proportions. Differences between continuous variables were tested using the Wilcoxon-rank sum test or the t-test and between categorical variables, with the Chi-squared or Fisher’s exact test.

The incidence rate of CAC in RA patients and control subjects is presented as person-years and compared using Poisson regression. Multivariable regressions were modeled to examine if any differences were independent of traditional cardiovascular risk factors, using backward elimination by blocks.

Yearly progression rate was compared among RA patients and controls with CAC >0 at baseline. Robust linear regressions were modeled to examine whether progression was greater in RA patients than in controls and if any association was independent of CAC score at baseline and traditional cardiovascular risk factors.

The associations between traditional risk factors and progression of CAC were explored and heterogeneity was tested between 'caseness’ and selected variables with multiplicative interaction models.

Finally, among RA patients, the association between traditional cardiovascular risk factors and RA disease characteristics with progression of CAC was also explored. Variables included disease activity, radiographic damage, medications and markers of inflammation. Poisson regression or robust linear regression was used, as appropriate.

All statistical tests were calculated using a 5% two-sided significance level using STATA/IC 11.0 (StataCorp, College Station, Texas, USA).

## Results

Table 1 presents the clinical characteristics at baseline of the 155 RA patients and the 835 MESA participants. Patients with RA were, on average, four years younger than control subjects. There were higher proportions of women, Caucasians and individuals who completed a 12th grade education in the RA group than in the control group (each *P* <0.05).

**Table 1:** Baseline characteristics according to RA status

**Characteristic**	**RA**	**Controls**	***P***
	**Number = 1 55**	**Number = 835**	
Demographics			
Age, years	59.2 ± 8.3	62.8 ± 10.0	<0.001
Female; n (%)	97 (63%)	437 (52%)	0.019
Caucasian race; n (%)	136 (88%)	425 (51%)	<0.001
Education; n (%)	122 (79%)	580 (70%)	0.035
Some college or higher			
Cardiovascular Risk Factors			
Diabetes; number (%)	11 (7%)	137 (16%)	0.003
Hypertension (%)	87 (56%)	465 (56%)	0.92
Systolic BP, mm Hg	126 ± 17	126 ± 20	0.72
Diastolic BP, mm Hg	75 ± 9	71 ± 10	<0.001
Dyslipidemia			
Total cholesterol, mg/dL	198 ± 40	192 ± 35	0.10
LDL cholesterol, mg/dL	117 ± 32	118 ± 31	0.74
HDL cholesterol, mg/dL	56 ± 20	51 ± 14	<0.001
Triglycerides, mg/dL	126 ± 98	117 ± 72	0.20
Cigarette smoking			
Current; number(%)	15 (10%)	103 (12%)	0.32
Serum inflammatory markers			
CRP, mg/L; median (IQR)	2.3 (1.1, 6.7)	2.2 (1.0, 4.6)	0.18
IL-6, pg/mL; median (IQR)	3.6 (1.6, 7.5)	1.3 (0.8, 1.91)	<0.001
Fibrinogen, mg/dL; median (IQR)	335 (278, 416)	339 (294, 387)	0.79
Prevalence of CAC	82 (52.9%)	454 (54.3%)	0.79
Unadjusted CAC score (Agatston Units)	3.1 (0, 135.1)	6.4 (0, 119.6)	0.68

BP, blood pressure; CAC, coronary artery calcium; CRP, C-reactive protein; HDL, high density lipoprotein; IQR, interquartile range; LDL, low density lipoprotein; RA, rheumatoid arthritis.

Patients and controls had a similar prevalence of hypertension, but diabetes at baseline was more prevalent in controls than in RA patients. Average HDL cholesterol was higher in RA participants than in controls, but there were no statistically significant differences in average LDL cholesterol or triglyceride levels. Not surprisingly, patients with RA had higher concentrations of IL-6, on average (*P* <0.001). However, RA patients did not have higher concentrations of CRP or fibrinogen, on average, compared to controls.

Patients with RA had a median disease duration of nine years and a mean DAS28 of 3.7 ± 1.1. Seventy percent had the shared epitope, 65% were seropositive for RF and 77% seropositive for anti-CCP antibodies. The median (IQR) Sharp score was 55 (16 to 120) and the median HAQ score was 0 (0 to 1).

The median baseline CAC scores were not statistically significantly different between the RA and control groups (median (IQR): 3.1 (0, 135.1) and 6.4 (0, 119.6) Agatston units, respectively, *P* = 0.68). However, in an analysis restricted to participants with baseline CAC scores >0 and adjusted for differences in cardiovascular disease (CVD) risk factors, baseline adjusted CAC scores were higher in patients with RA than control subjects (median (IQR): 173.6 (133.0 to 187.9) and 116.1 (88.5 to 145.7) Agatston units, respectively), (β = 52.3, *P* = 0.02). A sensitivity analysis in which RA (n = 86) and control (n = 86) participants were matched 1:1 for age, sex, race, diabetes, smoking and dyslipidemia demonstrated comparable findings. In these analyses, baseline prevalences of CAC in RA versus controls were 52.3% and 46.5%, respectively, *P* = 0.4; in those with baseline CAC scores >0, median CAC scores were (median (IQR)) 135 (21, 472) and 115 (27, 362) in RA and controls, respectively.

### Incident CAC

Table 2 presents the incidence rates of detectable CAC. Of the 73 patients with RA and a CAC score of zero at baseline, 20 (27%) developed detectable CAC during an average of 3.3 ± 0.3 years of follow-up, resulting in an incidence rate of 8.2 per 100 person-years. In the control group, there were 381 participants free of CAC at baseline. Among those, 65 (17%) developed detectable CAC over an average of 2.4 ± 0.9 years of follow-up, resulting in an incidence rate of 7.3 per 100 person-years.

**Table 2:** Incidence rate of CAC (among participants with no CAC at baseline)

	**RA**	**Controls**
	**(Number = 73)**	**(Number = 381)**
Incident CAC, number	20	65
Person-year	243	895
Incidence rate (100 person/year)	8.2	7.3
Unadjusted IRR	1.14 (0.73, 1.75)	
IRR - Model 1^a^	1.28 (0.83–1.98)	
IRR - Model 2^b^	1.02 (0.63, 1.65)	
IRR - Model 3^c^	1.11 (0.66, 1.88)	

^a^Adjusted for age; ^b^adjusted for age, gender, ethnicity, smoking, dyslipidemia, diabetes, and hypertension; ^c^interaction with gender (*P* for interaction = 0.56). CAC, coronary artery calcium; IRR, incidence rate ratio; RA, rheumatoid arthritis.

When both incidence rates were compared, the unadjusted incidence rate ratio (IRR) was 1.14 (95% CI 0.73 to 1.75). This result did not change substantially after adjustment for demographic variables and traditional cardiovascular risk factors. A sensitivity analysis, restricting controls with similar time to follow-up as RA patients, yielded similar results (data not shown).

### Progression of CAC

RA patients with any detectable CAC at baseline had a median yearly rate of progression of 21 (7 to 62) Agatston units. Although the IQR varied slightly, the median rate of progression was also 21 (5 to 70) Agatston units in control subjects. As shown in Table 3, there was no statistically significant association between progression of calcium scores and RA after adjustment for demographic variables and traditional cardiovascular risk factors. Sensitivity analyses in which controls were restricted to those who had similar time to follow-up as the RA patients, and another in which adjusted baseline CAC scores were entered into the mode, gave us similar results (data not shown). In a final sensitivity analysis in which RA patients (n = 86) were matched 1:1 to controls (n = 86) for age, sex, race, diabetes, smoking, and dyslipidemia, progression rates were not statistically different (19.3 (5.4 to 48.0) and 31.4 (4.2 to 66.5) Agatston units for RA and controls, respectively (*P* = 0.5).

**Table 3:** Yearly progression among participants with any CAC at baseline

	**RA**	**Controls**
Median yearly progression (Agatston units): median (IQR)	21 (7 to 62)	21 (5 to 70)
Coef (unadjusted)	0.5 (-8.2, 9.1)	
Coef - Model 1^a^	2.1 (-6.5, 10.7)	
Coef - Model 2^b^	1.4 (-7.5, 10.4)	
Coef - Model 3^c^	4.1 (-9.0, 17.3)	

^a^Adjusted for age; ^b^adjusted for age, gender, ethnicity, smoking, dyslipidemia, diabetes, and hypertension; ^c^with gender using interaction model. CAC, coronary artery calcium; IQR, interquartile range.

### Risk factors associated with incident coronary artery calcium

RA patients who developed newly detectable CAC were older (59+/-7 versus 55 ± 6 years old, *P* = 0.03), had higher concentrations of triglycerides at baseline (137 ± 86 versus 97 ± 60 mg/dl, *P* = 0.01) and higher systolic blood pressure (129 ± 17 versus 117 ± 15 mm Hg, *P* = 0.01) than those who did not develop any new coronary calcification. The interaction analyses did not show disease status heterogeneity by age, triglycerides or systolic blood pressure with regard to incident CAC (*P* values for interaction = 0.36, 0.25, and 0.11, respectively). The differences in triglycerides and blood pressure remained statistically significant after adjustment for age. There were no observed statistical differences in the concentration of inflammatory markers or disease characteristics at baseline or as average over follow-up among RA patients who developed coronary calcium and those who did not (Table 4).

**Table 4:** Baseline characteristics associated with incident CAC (among RA patients free of CAC at baseline)

	**Incident CAC: yes**	**Incident CAC: no**	***P*-value**	**IRR (95% CI)**	**Adjusted IRR**^**a **^**(95% CI)**
Demographics					
Age, years	59 ± 7	55 ± 6	0.03	1.06 (1.0, 1.14)	NA
Male; number (%)	5 (25%)	8 (15%)	0.32	1.65 (0.71, 3.84)	1.81 (0.79, 4.18)
Caucasian race; number (%)	19 (95%)	44 (83%)	0.18	3.03 (0.45, 20.7)	2.58 (0.39, 17.3)
Education; number (%)	16 (80%)	44 (83%)	0.76	0.86 (0.33, 2.21)	0.85 (0.32, 2.27)
Some college or higher					
Cardiovascular Risk Factors					
Diabetes; number (%)	1 (5%)	2 (4%)	0.81	1.14 (0.20, 6.46)	1.08 (0.21, 5.65)
Hypertension (%)	11 (55%)	21 (40%)	0.24	1.56 (0.73, 3.33)	1.42 (0.67, 3.0)
Systolic BP, mm Hg	129 ± 17	117 ± 15	0.01	1.03 (1.01, 1.05)	1.02 (1.0, 1.04)*
Diastolic BP, mm Hg	76 ± 9	72 ± 8	0.09	1.03 (0.99, 1.09)	1.05 (1.01, 1.09)*
Dyslipidemia (%)					
Total cholesterol, mg/dL	201 ± 29	192 ± 41	0.40	1.0 (0.99, 1.01)	1.0 (0.99, 1.01)
LDL cholesterol, mg/dL	116 ± 20	112 ± 34	0.66	1.0 (0.99, 1.01)	1.0 (0.99, 1.01)
HDL cholesterol, mg/dL	57 ± 16	61 ± 19	0.51	0.99 (0.97, 1.01)	0.98 (0.96, 1.01)
Triglycerides, mg/dL	137 ± 86	97 ± 60	0.03	1.0004 (1.0, 1.01)	1.004 (1.0, 1.01)*
Cigarette Smoking					
Current; number (%)	2 (10%)	4 (8%)	0.73	1.26 (0.36, 4.49)	0.97 (0.24, 3.94)
Serum inflammatory markers					
CRP, mg/L; median (IQR)	2.1 (0.5 to 9.6)	2.1 (0.8 to 5.2)	0.74	0.96 (0.72, 1.27)	0.99 (0.75, 1.32)
IL-6, pg/mL; median (IQR)	4.0 (1.2 to 7.7)	2.3 (1.1 to 7.5)	0.45	1.12 (0.79, 1.58)	1.17 (0.83, 1.64)
Fibrinogen, mg/dL; median (IQR)	362 (260 to 473)	338 (278 to 383)	0.89	1.08 (0.33, 3.50)	1.23 (0.39, 3.94)
E-selectin, ng/mL; median (IQR)	49 (25 to 76)	45 (29 to 59)	0.90	1.0 (0.53, 1.88)	1.02 (0.58, 1.80)
s-ICAM-1, ng/mL; median (IQR)	312 (247 to 383)	275 (225 to 322)	0.12	1.60 (0.58, 4.40)	1.31 (0.47, 3.62)
Disease characteristics at baseline					
Disease duration (years)	7 (4 to 14)	10 (5 to 20)	0.13	0.98 (0.94, 1.02)	0.97 (0.94, 1.01)
DAS28	3.9 ± 0.9	3.7 ± 1.0	0.44	1.19 (0.81, 1.75)	1.20 (0.81, 1.78)
HAQ	0.5 ± 0.7	0.5 ± 0.6	0.87	1.10 (0.60, 2.04)	1.12 (0.64, 1.95)
Sharp score; median (IQR)	29 (15 to 73)	44 (11 to 90)	0.93	1.0 (0.99, 1.0)	1.0 (0.99, 1.0)
Current use of MTX	10 (50%)	38 (72%)	0.08	0.52 (0.24, 1.09)	0.51 (0.25, 1.04)
Current use of biologic agents	11 (55%)	24 (45%)	0.46	1.30 (0.61, 2.79)	1.29 (0.62, 2.68)
Current use of corticosteroids	7 (35%)	19 (36%)	0.95	0.95 (0.43, 2.11)	1.02 (0.46, 2.26)
Disease characteristics, expressed as average over follow-up					
DAS28	3.5 ± 1.0	3.5 ± 1.0	0.90	1.04 (0.7, 1.6)	1.06 (0.7, 1.6)
CRP, mg/L; median (IQR)	2.0 (0.9 to 6.5)	2.2 (1.2 to 6.2)	0.72	1.0 (1.0, 1.1)	1.0 (1.0, 1.1)
IL-6, pg/mL; median (IQR)	4.7 (1.5 to 20.9)	3.7 (2.0 to 11.5)	0.87	1.0 (1.0, 1.2)	1.0 (1.0, 1.1)
Fibrinogen, mg/dL; median (IQR)	358 (283 to 425)	361 (310 to 419)	0.78	1.0 (1.0, 1.0)	1.0 (1.0, 1.0)
E-selectin, ng/mL; median (IQR)	44 (34 to 61)	43 (34 to 65)	0.78	1.0 (1.0, 1.0)	1.0 (1.0, 1.0)
s-ICAM-1, ng/mL; median (IQR)	327 (242 to 367)	266 (240 to 323)	0.12	1.0 (1.0, 1.0)	1.0 (1.0, 1.0)

^a^Adjusted for age. *P <0.05. BP, blood pressure; CAC, coronary artery calcium; CRP, C-reactive protein; DAS28, disease activity score in 28 joints; HDL, high density lipoprotein; IQR, interquartile range; IRR, incidence rate ratio; LDL, low density lipoprotein; MTX, methotrexate; RA, rheumatoid arthritis; s-ICAM, soluble intercellular adhesion molecule.

### Risk factors associated with progression of coronary artery calcium

Coronary calcium scores progressed at a higher rate in those patients with RA who were Caucasian and had lower triglyceride concentrations (Table 5). This latter association became of borderline significance after adjustment for age, sex, race, hypertension, diabetes and smoking (*P* = 0.08). No other associations were seen between patient characteristics and faster rates of progression of CAC in patients with RA.

**Table 5:** Clinical Characteristics and the Risk of Progression of CAC (among those RA patients with Detectable CAC at baseline)

***Demographics***	***B (95% CI)***	***Adjusted B***^**a**^ *** (95% CI)***
Age, years	-0.3 (-0.6, 1.3)	NA
Male sex	8.3 (-8.1, 24.6)	NA
Caucasian race; number (%)	28.0 (2.2, 53.7)	NA
Education; number (%)	-2.8 (-22.1, 16.5)	-2.3 (-24.3, 19.7)
Some college or higher		
Cardiovascular Risk Factors		
Diabetes; number (%)	-6.5 (-32.5, 19.5)	9.8 (-18.8, 38.4)
Hypertension (%)	13.1 (-2.3, 28.6)	5.4 (-14.6, 25.5)
Systolic BP, mm Hg	-0.3 (-0.8, 0.2)	-0.2 (-0.8, 0.4)
Diastolic BP, mm Hg	0.1 (-0.8, 1.0)	0.1 (-1.0, 1.2)
Dyslipidemia (%)	16.7 (-1.2, 34.6)	14.0 (-4.8, 32.8)
Total cholesterol, mg/dL	-0.1 (-0.3, 0.1)	-0.0 (-0.3, 0.2)
LDL cholesterol, mg/dL	0.0 (-0.3, 0.2)	0.1 (-0.3, 0.4)
HDL cholesterol, mg/dL	-0.1 (-0.4, 0.4)	0.2 (-0.4, 0.9)
Triglycerides, mg/dL	-0.03 (-0.1, 0.0)	-0.1 (-0.2, 0.0)
Cigarette Smoking		
Current; number (%)	19. 9 (-5.7, 45.5)	10.8 (-19.5, 41.2)
Serum inflammatory markers at baseline		
CRP, mg/L; median (IQR)	3.2 (-3.5, 9.9)	4.4 (-3.0, 11.7)
IL-6, pg/mL; median (IQR)	-1.8 (-11.4, 7.8)	-0.8 (-11.4, 9.9)
Fibrinogen, mg/dL; median (IQR)	19.0 (-10.6, 48.5)	13.9 (-20.0, 47.8)
Disease characteristics at baseline		
Disease duration (years)	0.4 (-0.4, 1.2)	0.6 (-0.28, 1.5)
RF	3.9 (-13.2, 21.0)	7.0 (-11.6, 25.6)
Anti-CCP	4.2 (-15.9, 24.4)	-1.5 (-25.5, 22.5)
SE	2.2 (-16.2, 20.6)	-6.8 (-27.8, 14.3)
DAS28	5.1 (-2.4, 12.7)	6.5 (-1.5, 14.5)
HAQ	-5.1 (-17.6, 7.4)	-0.8 (-16.5, 14.9)
Sharp score	-0.0 (- 0.1, 0.1)	-0.0 (-0.1, 0.1)
Current use of MTX	-2.9 (-19.6, 13.7)	-3.2 (-17.9, 23.2)
Current use of biologic agents	15.8 (-1.5, 33.0)	18.5 (-1.4, 38.4)
Current use of corticosteroids	-8.6 (-25.8, 8.1)	-5.3 (-26.1, 15.3)
Disease characteristics, expressed as average over follow-up		
DAS28	2.0 (-7.6, 11.6)	4.8 (-6.0, 15.5)
CRP	-3.9 (-11.2, 3.5)	-1.1 (-9.7, 7.5)
IL-6	-5.4 (-13.6, 2.9)	-6.0 (-16, 4.0)
Fibrinogen pg/mL; median (IQR)	-7.3 (-45, 29)	-6.1 (-50, 38)

^a^Adjusted for age, sex, race, hypertension, diabetes, smoking, dyslipidemia. BP, blood pressure; CAC, coronary artery calcium; CCP, cyclic citrullinated peptide; CRP, C-reactive protein; DAS28, disease activity score in 28 joints; HAQ, Health Assessment Questionnaire; HDL, high density lipoprotein; IQR, interquartile range; IRR, incidence rate ratio; LDL, low density lipoprotein; MTX, methotrexate; RA, rheumatoid arthritis; rheumatoid factor; RR, relative risk; SE, shared epitope.

## Discussion

To the best of our knowledge, this is the first study evaluating determinants of CAC incidence and progression in patients with RA. Our main findings can be summarized in three parts. First, our results indicate that there were no statistically significant differences in rates of incident CAC in patients and controls. Second, once patients have any CAC, the progression is similar to the progression seen in controls. Third, age, blood pressure and triglyceride concentrations, but not markers of inflammation or measures of disease activity/damage, predicted newly identified coronary calcium in RA.

Our analysis suggesting similar progression of CAC in patients and controls was not concordant with our primary hypothesis or with a prior study that showed greater progression of intima-media thickness (IMT) in the common carotid of RA patients compared to controls [26]. There are several potential explanations for this apparent discordance. First, as suggested by Maradit-Kremers *et al*., atherosclerosis may precede the clinical presentation of RA [27]. Thus, given that we focused on subclinical atherosclerosis, RA patients with prior events, who may have contributed to an even greater progression of CAC, had been excluded, and this could have introduced a differential bias. Second, it is likely that the increased CV event rate in RA patients compared to controls in epidemiologic studies is explained, at least in part, by rupture of vulnerable non-calcified plaque.

Many potential predictors of progression were explored in our analysis. Our exploratory results indicate that age, hypertriglyceridemia and blood pressure were associated with incident CAC but we did not find statistically significant associations with RA-specific variables. In contrast, a recent analysis by our group indicated that higher swollen joint counts and cumulative average CRP predicted progression of carotid plaque in patients with RA [28]. While both carotid ultrasound (US) and CAC scores ascertain subclinical atherosclerosis, CAC measures only calcified plaque while carotid US measures calcified and non-calcified plaque as well as intima media thickness (IMT). Thus, each of these measures provides different information. Calcified plaques are good predictors of myocardial infarction (MI) and CVD mortality events in the general population, but they are more stable, and thus, less prone to rupture and cause an event.

In contrast, carotid US measures both non-calcified and calcified plaques. Non-calcified plaques account for approximately three-quarters of all coronary lesions [29], are associated with inflammation [30] and are also important predictors of cardiovascular hard events [31]. Thus, it is possible that the assessment of this type of plaque could improve cardiovascular risk stratification in patients with RA. This study has several strengths. First, it compares two contemporary cohorts and data were collected prospectively. Second, both groups were studied following similar and rigorous protocols. Third, it uses state of the art techniques to ascertain CAC and to measure inflammatory markers.

Our study also has some limitations. First, although the rate of successful follow-up was high, 20% of patients with RA either declined or could not be contacted for their final follow-up assessment. We hypothesized that patients who were lost to follow-up may have had more severe disease; however, median baseline DAS and HAQ scores were similar among patients who came back for a second visit and those who were lost to follow-up. Second, the average follow-up was only 3.2 years in RA patients and 2.3 years in controls and, to account for this, required estimation of average yearly progression. Longer follow-up and the evaluation of CV events, such as acute MI and CV deaths, will be more informative. Third, type II error might explain lack of statistical significance in some of the subgroup analyses. For example, a pre-study power analysis estimated that to show a difference in CAC scores among individuals with any coronary calcification detected at baseline would have required a difference of 26 Agatston units per year between RA patients and controls. In addition, a younger cohort of patients with lower burden of coronary atherosclerosis at baseline and higher RA disease activity could have increased the likelihood of a positive result. Fourth, the lack of association between RA and progression of subclinical coronary atherosclerosis does not exclude the possibility of differences in progression of arterial calcification in other vascular beds, such as thoracic aorta.

## Conclusions

In summary, the incidence and progression of coronary calcium did not differ significantly between RA and non-RA groups. In patients with RA, higher systolic blood pressure, higher triglycerides concentrations and older age were significant predictors of incident CAC over the period of follow-up, while inflammatory and RA disease characteristics were not. Among patients with RA and CAC at baseline, no association was found between traditional risk factors and CAC progression.

## Abbreviations

BP: Blood pressure; CAC: Coronary artery calcium; CCP: Cyclic citrullinated peptide; CRP: C-reactive protein; CT: Computerized tomographic; CV: Cardiovascular; CVD: Cardiovascular disease; DAS: Disease activity score; ESCAPE RA: Evaluation of subclinical cardiovascular disease and predictors of events in rheumatoid arthritis; HAQ: Health assessment questionnaire; HDL: High density lipoprotein; HLA: Human leukocyte antigen; IL-6: Interleukin-6; IMT: Intima media thickness; IQR: Interquartile range; IRR: incidence rate ratio; LDL: Low density lipoprotein; MDCT: Multidetector row computed tomography; MESA: Multiethnic study of atherosclerosis; MI: Myocardial infarction; RA: Rheumatoid arthritis; RF: Rheumatoid factor; US: Ultrasound.

## Competing interests

The authors declare that they have no competing interests.

## Authors’ contributions

CPC participated in analysis and interpretation of the data and drafting and revising the manuscript. JTG participated in design of the study, acquisition of the clinical data, analysis and interpretation of the data and reviewing and revising the manuscript. RK participated in the statistical design, analysis and interpretation of the data and reviewing and revising the manuscript. WSP participated in design of the study, acquisition of the carotid ultrasound data and reviewing and revising the manuscript. ACG participated in the analysis and interpretation of the data and reviewing and revising the manuscript. MP participated in the analysis and interpretation of the data and reviewing and revising the manuscript. MS participated in the design of the study, analysis and interpretation of the data and reviewing and revising the manuscript. RD participated in acquisition of the coronary artery calcium data, reviewing and revising the manuscript and gave final approval of the data. MJB participated in acquisition of the coronary artery calcium data, reviewing and revising the manuscript and gave final approval of the data. RSB participated in design of the study, acquisition of the coronary calcium data and reviewing and revising the manuscript. PO participated in the analysis and interpretation of the data and reviewing and revising the manuscript. DB participated in acquisition of the coronary calcium data and reviewing and revising the manuscript. JMB designed the study, participated in acquisition of all aspects of data, participated in analysis and interpretation of data and drafted and revised the manuscript. All authors read and approved the final manuscript.
